# Oral Health-Related Quality of Life of Hong Kong Kindergarten Children Receiving Silver Diamine Fluoride Therapy

**DOI:** 10.3390/dj12080248

**Published:** 2024-08-05

**Authors:** Hollis Haotian Chai, Ivy Guofang Sun, Duangporn Duangthip, Sherry Shiqian Gao, Edward Chin Man Lo, Chun Hung Chu

**Affiliations:** 1 Faculty of Dentistry, The University of Hong Kong, Hong Kong 999077, China; htchai89@connect.hku.hk (H.H.C.); ivysun@connect.hku.hk (I.G.S.); duangthip.2@osu.edu (D.D.); sherrysgao@xmu.edu.cn (S.S.G.); hrdplcm@hku.hk (E.C.M.L.); 2 Division of Pediatric Dentistry, The Ohio State University, Columbus, OH 43210, USA; 3 Department of Stomatology, School of Medicine, Xiamen University, Xiamen 361005, China

**Keywords:** oral health-related quality of life, OHRQoL, silver diamine fluoride, caries, early childhood caries, children

## Abstract

The objective of this prospective 12-month observational study is to examine the oral health-related quality of life (OHRQoL) among Hong Kong young children aged 3–4 years old receiving silver diamine fluoride (SDF) therapy for carious upper anterior primary teeth. A parental questionnaire was used to collect each child’s sociodemographic background and oral health habits at baseline. Data on parents’ satisfaction with their child’s dental aesthetics were collected at baseline and during a 12-month visit. The Chinese Early Childhood Oral Health Impact Scale (C-ECOHIS) was used to measure OHRQoL. A trained dentist performed examinations and recorded caries experience (dmft) and oral hygiene (visible plaque index) at baseline and 12 months. SDF was applied to the carious lesions. Out of 286 invited children, 248 (87%, 248/286) participated, and 211 (85%, 211/248) attended the 12-month examination. All SDF-treated carious lesions were discoloured at the 12-month examination. Regression analysis showed that the baseline C-ECOHIS score was associated with dmft (*p* < 0.001). The baseline and 12-month C-ECOHIS scores were 4.6 ± 5.5 and 5.0 ± 5.6, respectively (*p* = 0.42). The scores for parental satisfaction with dental aesthetics at baseline and 12 months were 59% to 46% (*p* < 0.001). Satisfaction was negatively associated with the number of discoloured upper anterior teeth (*p* < 0.001). In conclusion, SDF discoloured the carious upper anterior teeth of the Hong Kong kindergarten children. However, the discoloured lesions had no significant effects on the OHRQoL of these children. However, more parents became dissatisfied with their child’s dental aesthetics after SDF therapy. Hence, clinicians should inform parents well before they perform SDF therapy on children.

## 1. Introduction

Early childhood caries (ECC) is defined as the presence of one or more decayed, missing (due to caries) or filled surfaces in any primary tooth among children under the age of six [1]. ECC remains a significant chronic disease of childhood and public health problems, affecting almost half of preschool children globally [2]. The consequences of ECC extend beyond children’s oral health, affecting their overall growth, including their psychological well-being, and placing a burden on their families [3]. In clinical practise, oral health status is typically assessed using clinical parameters, including oral hygiene and caries experience. While these clinical parameters are undoubtedly crucial for measuring oral health status in preschool children, it is important to recognise that numerous physical and psychosocial issues cannot be determined solely by these clinical parameters [4]. Consequently, numerous instruments have been developed to evaluate preschool children’s functional well-being, emotional well-being, social interactions, expectations and satisfaction with respect to their oral health, collectively referred to as Oral Health-Related Quality of Life (OHRQoL) [5]. Measuring OHRQoL for children in clinical practise is important for monitoring their responses to treatment, supporting overall development and promoting lifelong healthy habits [6]. This assessment aids dental professionals and caregivers in providing appropriate care and support, fostering improved oral health and well-being for young patients [7]. Several tools are available to assess the OHRQoL of preschool children. However, due to the variations in psychosocial awareness and cognitive abilities during childhood, selecting an appropriate measurement that accurately captures the subjective feelings of children presents challenges [8]. Researchers used the Early Childhood Oral Health Impact Scale (ECOHIS) to assess OHRQoL for preschool children [9]. ECOHIS derives its data from a 13-item parental administrated questionnaire. A Chinese version of this scale, known as the C-ECOHIS (Chinese version Early Childhood Oral Health Impact Scale), has been translated and validated [10].

In Hong Kong, the government has been promoting oral health for young children through oral health education. Oral health education programmes like Brighter Smiles Playland have been developed for kindergarten students [11]. The government has also fluoridated the water supply at 0.5 ppm [12], and fluoride toothpaste is available at an affordable cost. However, the prevalence of ECC among 5-year-old children is approximately 50%, and more than 90% of ECCs are left untreated [13]. Conventional restorative care is often unaffordable, unavailable and inaccessible to many children. The World Health Organisation (WHO) has recently included silver diamine fluoride (SDF) in the WHO Model Lists of Essential Medicine [14]. SDF has gained attention in ECC management as an effective, non-invasive and painless agent [15]. Recently, an outreach oral health promotion project was introduced to all kindergartens in Hong Kong. This initiative aims to improve the oral health of preschool children by preventing and controlling ECC through the adoption of SDF therapy. Participating children receive an annual dental examination, oral health education and SDF treatment, all free of charge. Following the dental examination, parents are provided with an oral health report detailing their child’s oral health status [16]. 

A review concluded that ECC affects all primary teeth, with the upper anterior teeth being predominantly affected [17]. The potential aesthetic issue arising from SDF black discolouration might contribute to parental concerns regarding the acceptance of this treatment option [18]. A literature search found limited information available on the OHRQoL of young children following the use of SDF in school-based oral health promotion programmes. Only one study has assessed the changes in OHRQoL among preschool children after receiving SDF therapy for six months. This 6-month study focused on 4- to 5-year-old preschool children with dental caries, irrespective of caries position [19]. The objective of this prospective 12-month observational study is to examine the oral health-related quality of life (OHRQoL) among Hong Kong young children aged 3–4 years old receiving silver diamine fluoride (SDF) therapy for carious upper anterior primary teeth. The primary outcome of this study was to investigate the change in OHRQoL among 3- to 4-year-old children participating in the kindergarten-based oral health promotion project who had caries in their upper anterior teeth, after receiving SDF therapy for 12 months. The secondary outcome was to explore the potential factors associated with OHRQoL in the studied population.

## 2. Materials and Methods

This observational study used C-ECOHIS to measure the OHRQoL of Hong Kong young children attending a kindergarten-based oral health promotion project. This study was conducted from December 2019 to May 2021. Ethics approval was obtained from the Institutional Review Board of the University of Hong Kong/Hospital Authority Hong Kong West Cluster (UW 19-660).

### 2.1. Participants

The sample size calculation for this study was conducted using G*Power 3.1.9.6 (University of Düsseldorf, Düsseldorf, Germany). Based on previous research findings [19], the minimal important difference (MID) of the mean C-ECOHIS score before and after was set at 1.5, and the standard deviation (SD) of the mean C-ECOHIS score at baseline was set at 6.6. The study’s power was established at 90% (β = 0.10), and a two-sided test with a statistical significance level of 0.05 was employed. Considering a projected 15% dropout rate for the follow-up examination and an anticipated 85% response rate for the kindergarten-based oral health promotion programme, we concluded that a minimum of 285 children should be invited as the baseline for this study. 

In this study, kindergarten children were recruited from three primary districts: Hong Kong Island, Kowloon, and the New Territories. A stratified random sampling method was employed to ensure a representative sample of participants from each district. The parents received invitation letters outlining the purpose and methods of the study. Prior to the data collection, written parental approval was requested and obtained. Eligible participants included preschool children aged 3 to 4 years old who had developed at least one dentin caries in their upper anterior teeth, who had never previously received SDF treatment, who were generally healthy, whose parents or guardians were able to read and write in Chinese, and who cooperated with the dental examination. Being recalcitrant, refusing to be examined, and having serious systemic disorders were among the exclusion criteria.

### 2.2. Clinical Assessment

Each child received a dental examination by one calibrated dentist at baseline and a 12-month follow-up. The clinical diagnosis of oral hygiene and caries status was completed through visual inspection using disposable dental mirrors, WHO community periodontal index dental probes (405/WHO probe; Otto Leibinger; Mühlheim; Germany) and headlamps. Caries status was recorded using the dmft index following WHO’s recommendation as decayed, missing and filled teeth (dmft). When a carious lesion felt soft upon gentle probing, it was recorded as active; when it felt hard upon gentle probing, it was recorded as arrested. Oral hygiene status was recorded using the virtual plaque index (VPI). The buccal and lingual surfaces of the six index teeth (55, 51, 63, 71, 75 and 83) were assessed to examine the presence or absence of visual dental plaque. The presence of dental plaque was recorded as 1, and its absence was recorded as 0. The odds of the number of surfaces with dental plaque present divided by the total number of examined surfaces was used to represent oral hygiene status. The VPI score ranged from 0 to 1. The study children were examined and followed up in their kindergartens with the help of the teachers. A duplicate examination was conducted on approximately 10% of the recruited children in every visit to assess the intra-examiner agreement. Participating children with dentin caries were treated with 38% silver diamine fluoride (SDF) (Saforide, Toyo Seiyaku Kasei Co., Ltd., Japan) at baseline. After the clinical examination, each child’s parents received a personalised report on the state of the child’s dental health.

### 2.3. Parental Questionnaires 

The parental questionnaires were collected at baseline and during a 12-month follow-up. A parental questionnaire was used to collect each child’s sociodemographic background (sex, parental education level, family income), snacking habits, bottle feeding habits and tooth-brushing habits at baseline. Parents were asked if they were satisfied with their child’s dental aesthetics at baseline and during the final visit. C-ECOHIS was used to measure the OHRQoL of the preschool children. It comprises thirteen items with the child impact section (CIS, nine items) and the family impact section (FIS, four items) [10]. The CIS has four domains (one item for symptoms, four items for function, two items for psychological factors, and two items for social interaction). The FIS has two domains (two items for parental distress, two items for family function). The possible response to each item was scored on a 5-point scale: 0 = never; 1 = hardly ever; 2 = occasionally; 3 = often; 4 = very often; 5 = do not know. A simple sum of the response codes was used to determine the overall C-ECOHIS scores as well as the scores for each domain. Possible scores ranged from 0 to 36 for CIS, 0 to 16 for FIS and 0 to 52 for total C-ECOHIS, with higher scores signifying higher levels of oral impact on the child’s OHRQoL.

### 2.4. Statistical Analysis 

The original data from hard copies were input into Excel software and double-checked. The data were cleaned and then transferred to SPSS software (V29.0, Chicago, IL, USA) to conduct further statistical analysis. Descriptive statistics (numbers, percentages, means and standard deviations) were used to present data on the social demographic background, oral health habits, and oral health status of young children. To explore the relationships between these potential factors and the baseline C-ECOHIS score, bivariate analyses were conducted. Since the data were not normally distributed, Mann–Whitney U tests were employed to compare the distribution of C-ECOHIS scores among different groups. Additionally, Spearman’s rank correlation coefficients were utilised to examine the association between C-ECOHIS scores, dmft and VPI scores. To investigate the potential indicators associated with OHRQoL in the studied population, a negative binomial regression analysis was employed. Factors that were found to be significant in the bivariate analyses were included in the regression model. The incidence rate ratio (IRR) was estimated for each indicator. 

By deducting the post-treatment score (T1) from the pre-treatment score (T0), changes in the C-ECOHIS (T0–T1) scores were determined. If the post-treatment C-ECOHIS score was greater than the pre-treatment score (T0–T1 < 0), it indicated a negative change or a deterioration in OHRQoL, whereas a positive change or an improvement was suggested if the post-treatment score was lower than the pre-treatment score (T0–T1 > 0). A paired t-test was used to compare the total C-ECOHIS score before and after SDF therapy. The McNemar test was used to compare parental satisfaction with each child’s dental aesthetics before and after SDF therapy. The Mann–Whitney U tests was employed to explore the relationship between parental satisfaction with each child’s dental aesthetics after SDF therapy (satisfied or non-satisfied) and the number of discoloured upper anterior teeth for each child. The significance level of all statistical tests was set at 0.05.

## 3. Results

This study initially invited 286 children, of which 248 children completed valid questionnaires and received parental consent and received SDF therapy at baseline (response rate 87%, 248/286). Reasons for not receiving SDF therapy included children having other dental schemes or dentists, and concerns regarding staining. After 12 months, 211 out of the 248 children were followed up. The attrition rate was 15% (211/248). Out of the initial sample, 37 children did not undergo a 12-month dental examination. The baseline dmft values of these 37 children showed no statistically significant difference compared to the remaining participants in the study (*p* = 0.07). The children who had received dental examination and for whom the parental questionnaire had been completed at both baseline and 12 months were included for further data analysis. All SDF-treated carious lesions (*n*= 536) were found to be discoloured at final examination. 

The social demographics of the children and their oral health-related characteristics are shown in Table 1. The mean age of the participating children was 4.0 (0.6), and 52% of them were males. Within the sample of 211 children, 41% of the mothers held a tertiary education level (≥14 years). Similarly, 41% of the fathers had also achieved a tertiary education level (≥14 years). Nearly half (46%) of the children in the study belonged to families with an income below the median level. Their tooth-brushing habits showed that 43% of the participating children brushed their teeth less than 2 times a day. A total of 80% of the children had daily snacking habits. Additionally, 31% of the children exhibited bottle-feeding habits. At baseline, the mean (SD) values for dmft and VPI were 3.9 (3.0) and 0.5 (0.2), respectively. Table 1 illustrates the results of the bivariate analysis comparing the distribution of baseline C-ECOHIS scores among different groups. Among the examined variables, a significant difference was only observed in daily snacking habits. Children with daily snacking habits had higher baseline C-ECOHIS scores compared to those who did not (*p* = 0.007). Furthermore, caries experience (dmft) exhibited a positive correlation with the baseline C-ECOHIS score, as indicated by the Spearman’s rank correlation coefficients (rs = 0.289, *p* < 0.001).

To determine the impact of the identified factors on the baseline C-ECOHIS score, daily snacking and caries experience (dmft) were incorporated into a negative binomial regression model based on the bivariate analysis results. In the final regression model (Table 2), it was revealed that children with higher dmft scores had significantly higher C-ECOHIS scores (IRR = 1.12, *p* < 0.001).

**Figure 1:**
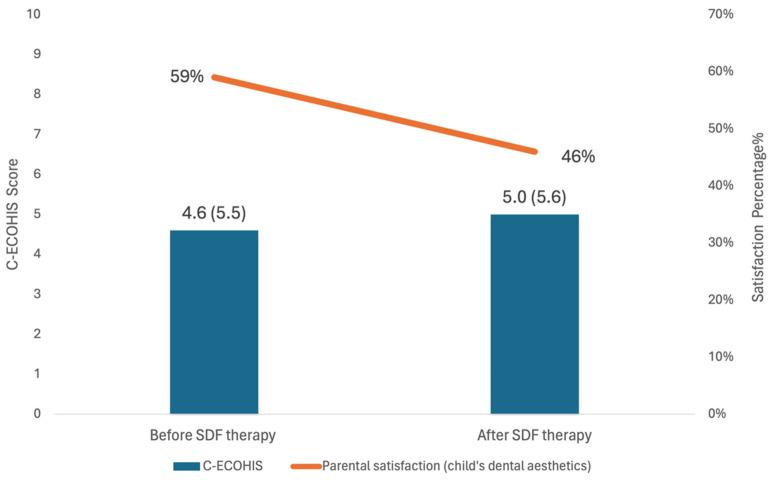
OHRQoL and parental satisfaction before and after SDF therapy.

At the baseline examination, the mean score (SD) for C-ECOHIS was 4.6 (5.5), CIS was 2.8 (3.9), and FIS was 1.8 (2.3). At the 12-month examination, the mean score (SD) for C-ECOHIS was 5.0 (5.6), CIS was 2.6 (3.1), and FIS was 2.4 (3.1). The difference in the mean score (SD) of C-ECOHIS between the two examinations was −0.4 (6.3) (Figure 1). The paired *t*-test revealed no significant differences in C-ECOHIS at baseline and during the 12-month examination (*p* = 0.423) pre- and post-SDF. The effect size was −0.06.

At baseline and the final examination, parental satisfaction with their child’s dental aesthetics was 59% (125/211) and 46% (98/211), respectively. The McNemar test revealed a statistically significant decline in parental satisfaction with the child’s dental aesthetics (*p* < 0.001) after the SDF treatment. The Mann–Whitney U test revealed a significant association between parental satisfaction regarding their child’s dental aesthetics (satisfied or unsatisfied) and the number of discoloured upper anterior teeth in the children (*p* < 0.001). Parents with children who have a greater number of discoloured upper anterior teeth are more likely to be unsatisfied with their child’s dental appearance.

## 4. Discussion

This study aimed to examine the change in OHRQoL among young children with caries in their upper anterior teeth who were part of a kindergarten-based oral health promotion project after undergoing SDF therapy for a period of 12 months. A paired *t*-test was used to compare the change before and after SDF therapy for the total C-ECOHIS score, even though the change in the OHRQoL score did not follow a normal distribution. Because the sample size was more than 200, which was large enough, the skew of the outcome (change) was minimal, and the distribution was approximately symmetric. The parametric tests were still robust with respect to violations of the normality assumptions, and were thus performed [19,20]. 

This study discovered that there was no change in OHRQoL for young children after undergoing SDF therapy for 12 months. The overall mean (SD) ECOHIS scores before and after SDF treatment were 4.6 (5.5) and 5.0 (5.6), respectively. This suggests that the overall influence of oral diseases and dental treatment experiences on Hong Kong’s young children was relatively low, both before and after SDF treatment. A prior observational study examining the association between OHRQoL and caries experience in Hong Kong preschool children also found that the overall impact of oral health was not substantial (scoring 5 out of 52). This, in conjunction with the present study, confirms the OHRQoL status of Hong Kong preschool children [21]. 

The current study’s findings regarding the impact of SDF treatment align with the previous investigation conducted on Hong Kong preschool children aged 4–5 years old, which assessed changes in OHRQoL following six months of SDF therapy [22]. Both studies observed no significant differences in the C-ECOHIS scores before and after SDF treatment, suggesting that the OHRQoL of Hong Kong preschool children aged 3–5 years remains stable after short-term (six months) and long-term (12 months) non-invasive treatment. The mean C-ECOHIS and dmft scores in the present study were lower than those in the aforementioned study, with mean C-ECOHIS and dmft scores of 4.6 (5.5) and 3.9 (3.0), respectively, compared to the previous study’s mean C-ECOHIS score of 7.4 (6.6) and dmft score of 4.9 (3.8). This discrepancy may be attributed to the younger age of children in the current study compared to the previous study (4.0 years old vs. 4.6 years old). 

Similar outcomes have been reported in studies conducted in other regions, such as Canada [23]. A previous review by Dr. Ruff and colleagues demonstrated that there was no significant difference in OHRQoL among children receiving SDF compared to other standard-of-care treatments [24]. The present study contributes additional evidence by examining within-subject changes in OHRQoL before and after SDF therapy for dental caries. Future longitudinal research on changes in OHRQoL both within SDF treatment and in comparison to other treatments would facilitate a more comprehensive understanding of the impact of these interventions on young children’s quality of life.

The results of the current study also revealed that more parents expressed dissatisfaction with their child’s dental aesthetics following SDF therapy. This dissatisfaction was found to be associated with the number of discoloured upper anterior teeth in the children. The increased visibility of caries lesions following SDF application might have led parents to perceive the darkened tooth surfaces as negatively affecting their child’s appearance. Prior studies have demonstrated that parents find it more acceptable to have the staining effect of arrested lesions in primary teeth applied to posterior teeth as opposed to anterior teeth [25].

Another possible explanation for their dissatisfaction could be that parents had unrealistic expectations regarding the outcomes of SDF treatment, resulting in disappointment when the actual results did not meet their initial expectations. This underlines the importance of providing clear and accurate information to parents about the anticipated outcomes and limitations of SDF therapy to manage parental expectations [26]. 

SDF is considered a safe, cost-effective and non-invasive treatment option for caries management. The American Academy of Pediatric Dentistry endorses the use of SDF as part of a caries management plan [27]. Additionally, the WHO has included SDF in its Model List of Essential Medicines for both adults and children [28]. Although an increase in parental dissatisfaction was observed, the stable OHRQoL indicates that SDF treatment remains an effective approach for managing dental caries in young children. This stability in OHRQoL suggests that the treatment provides adequate caries control, thereby maintaining the child’s oral health. 

Previous research has also suggested that the staining of dental decay resulting from SDF treatment does not appear to negatively impact OHRQoL [24]. To further understand parental perspectives regarding SDF treatment and their concerns about acceptance, qualitative research is necessary. This type of study would provide valuable insights into the reasons behind parents’ perceptions, allowing for a more comprehensive evaluation of SDF treatment and its implications on children’s oral health [29]. 

This study investigated the changes in OHRQoL for young children following the application of SDF as part of a kindergarten-based oral health promotion project. The SDF treatment was administered in a school setting, with participating children accompanied by their teachers. OHRQoL serves as a crucial evaluation indicator for assessing the performance of school-based dental programmes [30]. The OHRQoL of Hong Kong kindergarten children demonstrated its stability after SDF therapy in our study. 

There is limited information available on changes in OHRQoL following participation in community-based oral health programmes. The current study offers valuable insights to dental professionals regarding the application of SDF for young children in a school setting. This information enables oral health professionals to make informed decisions about appropriate care and treatment when implementing school-based oral health programmes. 

Certain limitations of this study should be noted. Firstly, the study population was restricted to Hong Kong, which may affect the generalisability of the findings. Secondly, the study design did not include a control group due to ethical considerations, which may introduce bias to the results. It is noteworthy that 13% of the invited children did not participate and 15% of the participating children failed to attend the 12-month examination. Despite these limitations, this study contributes important information to our understanding of SDF treatment in the context of school-based oral health programmes.

## 5. Conclusions

The OHRQoL of Hong Kong kindergarten children had no significant change after they underwent SDF therapy for 12 months, although SDF discoloured their carious upper anterior teeth. However, more parents were dissatisfied with their child’s dental aesthetics after SDF therapy.

## Figures and Tables

**Table 1:** Baseline C-ECOHIS scores of the studied young children according to their social demographic background, oral health habits and oral health status (*n* = 211).

Social Demographic Background	No. (%)	Mean C-ECOHIS (SD)	*p* Value ^1^
Age			0.202
3 years old	93 (44)	3.9 (4.8)	
4 years old	118 (56)	5.2 (6.0)	
Sex			0.692
Female	101 (48)	4.4 (5.7)	
Male	110 (52)	4.8 (5.5)	
Mother had tertiary education			0.789
Yes	87 (41)	4.2 (4.9)	
No	124 (59)	4.9 (6.0)	
Father had tertiary education			0.970
Yes	86 (41)	4.4 (5.0)	
No	125 (59)	4.8 (5.9)	
Family income above the median			0.452
Yes	114 (54)	4.3 (5.2)	
No	97 (46)	5.0 (5.9)	
**Oral health habits**	**No. (%)**	**Mean C-ECOHIS (SD)**	***p* Value ^1^**
Daily tooth brushing habits			0.283
<2 times/day	91 (43)	4.2 (5.0)	
≥2 times/day	120 (57)	5.0 (6.0)	
Daily snacking habits			*0.007*
Yes	169 (80)	5.0 (5.7)	
No	42 (20)	3.0 (4.5)	
Bottle-feeding habits			0.627
Yes	65 (31)	4.5 (5.7)	
No	146 (69)	4.7 (5.5)	
**Oral health status**	**Mean (SD)**	**Spearman’s rank** **correlation coefficient**	***p* Value ^2^**
Caries experience (dmft)	3.9 (3.0)	0.289	*<0.001*
Oral hygiene (VPI)	0.5 (0.2)	−0.085	0.220

^1^ *p* value was computed using Mann–Whitney U test. ^2^ *p* value was computed using Spearman’s rank correlation coefficient.

**Table 2:** Baseline C-ECOHIS score and selected independent variables (*n* = 211).

Items	Incidence Rate Ratio	95% CI	*p* Value
Daily snacking habits			
Yes	1.66	0.96–2.85	0.069
No ^a^			
Caries experience (dmft)	1.12	1.07–1.18	*<0.001*

Negative binomial regression; ^a^ reference group.

## Data Availability

The datasets generated and/or analysed during the current study are available from the corresponding author upon reasonable request.
